# Preferred binding of gain-of-function mutant p53 to bidirectional promoters with coordinated binding of ETS1 and GABPA to multiple binding sites

**DOI:** 10.18632/oncotarget.1708

**Published:** 2014-01-02

**Authors:** Catherine A. Vaughan, Swati P. Deb, Sumitra Deb, Brad Windle

**Affiliations:** ^1^ Integrative Life Sciences, Virginia Commonwealth University, Richmond, VA; ^2^ Department of Internal Medicine, Division of Hematology, Oncology and Palliative Care, Virginia Commonwealth University Richmond, VA; ^3^ Department of Biochemistry & Molecular Biology, Virginia Commonwealth University, Richmond, VA; ^4^ Massey Cancer Center, Virginia Commonwealth University, Richmond, VA

**Keywords:** mutant p53, ChIP-Seq, ETS1, bi-directional promoter

## Abstract

Gain-of-function mutant p53 is thought to induce gene expression in part by binding transcription factors bound to promoters for genes that mediate oncogenesis. We investigated the mechanism of mutant p53 binding by mapping the human genomic binding sites for p53 R273H using ChIP-Seq and showed them to localize to ETS DNA sequence motifs and locations with ETS1 and GABPA binding, both within promoters and distal to promoters. Strikingly, p53 R273H showed statistically significant and substantial binding to bidirectional promoters, which are enriched for inverted repeated ETS DNA sequence motifs. p53 R273H exhibited an exponential increase in probability of binding promoters with a higher number of ETS motifs. Both ETS1 and GABPA also showed an increase in the probability of binding to promoters with a higher number of ETS motifs. However, despite this increase in probability of binding by p53 R273H and ETS1, there was no increase in the binding signal, suggesting that the number of ETS1 and p53 R273H proteins bound per promoter is being limited. In contrast, GABPA did exhibit an increase in binding signal with higher numbers of ETS motifs per promoter. Analysis of the distance between inverted pairs of ETS motifs within promoters and binding by p53 R273H, ETS1 and GABPA, showed a novel coordination of binding for the three proteins. Both ETS1 and p53 R273H exhibited preference for binding promoters with distantly spaced ETS motifs in face-to-face and back-to-back orientations, and low binding preference to promoters with closely spaced ETS motifs. GABPA exhibited the inverse pattern of binding by preferring to bind promoters with closely spaced ETS motifs. Analysis of the helical phase between ETS motifs showed that ETS1 and p53 R273H exhibited a low preference for binding promoters with ETS motifs on the same face of the DNA helix. We propose a model for the binding of ETS1 and p53 R273H in which two inverted ETS motifs on a looped DNA helix are juxtaposed for ETS1 binding as a homodimer, with p53 R273H bound to ETS1. We propose that the formation of this DNA loop and protein-bound complex prevents additional binding of ETS1 and p53 R273H proteins to other proximal binding sites.

## INTRODUCTION

Mutations in the p53 gene are a significant contributor to most cancers. Approximately, 50% of cancers have p53 mutations and some cancer types, such as lung cancer, can reach a mutation rate as high as 70% [http://p53.free.fr/]. The missense mutations do not merely knockout p53 tumor suppressor activity but in many cases, also activate a dominant set of oncogenic functions. These functions represent a gain-of-function (GOF) for p53 that include increased growth rate, reduced apoptotic rate, increased motility, increased drug resistance, and increased tumorigenicity [1, 2].

While much of wild-type p53's functions are dependent on its sequence-specific transcriptional activation capability, the mechanism behind GOF is much less defined [1–5]. Like wild-type p53, gain-of-function (GOF) mutant p53 mediates changes in gene expression, however, the network of genes regulated by GOF mutant p53 is quite distinct from that for wild-type p53. The genes regulated by GOF mutant p53 are associated with oncogenic processes that include changes in the cell growth, cell cycle, invasion, metastasis, DNA replication, signal transduction, and survival pathways [1, 2, 6–8].

Proposed mechanisms for how GOF mutant p53 mediates changes in gene expression span a broad range of processes. Mutant p53 has been shown to interact with the p53-related proteins, p63 and p73 [9], and is proposed to effect changes in gene expression through perturbation of transcription regulation by p63 and p73 [1, 10]. Other transcription factors have been shown to interact with mutant p53 that include SP1 [11], ETS1 [12, 13], ETS2 [12], NF-Y [14], and the Vitamin D receptor [15]. Mutant p53 has been proposed to alter the regulation of genes regulated by these factors. It's interesting that SP1 and ETS1 are known interactors with wild-type p53 as well [16, 17]. For ETS1, GOF mutant p53 might in part induce oncogenic processes attributed to the proto-oncogene ETS1. Some studies have identified promoter sequences where GOF p53 has been shown to interact as identified by chromatin immunoprecipitation (ChIP), ChIP-Seq, ChIP on chip methods, presumably through binding to transcription factors that target these promoters [12, 14, 15, 18–21].

Our focus has been on how GOF mutant p53 interacts with the genome, the role for ETS1, and mechanism of gene activation. Our analysis of genomic binding characteristics for GOF mutant p53 and ETS1 has revealed a coordination of binding to multiple binding sites and a potential role for the ETS family factor, GABPA.

## RESULTS

### GOF mutant p53 interaction with the genome

We investigated the mechanisms of gene regulation mediated by GOF mutant p53 by analyzing how GOF mutant p53 (R273H) interacted with genomic sites when expressed in the human p53 null cell line, H1299, using ChlP-Seq. We initially focused our analysis of binding around known gene promoter regions by detecting specific binding signal for GOF mutant p53 for each 1Kb promoter region for RefSeq genes within H1299 expressing p53 R273H in comparison to the background signal from H1299 expressing no p53 (vector control). There were 812 promoter regions with significant binding specific to GOF mutant p53 determined for further analysis (Supplemental Table 1).

### p53 R273H binding specificity

**Figure 1:**
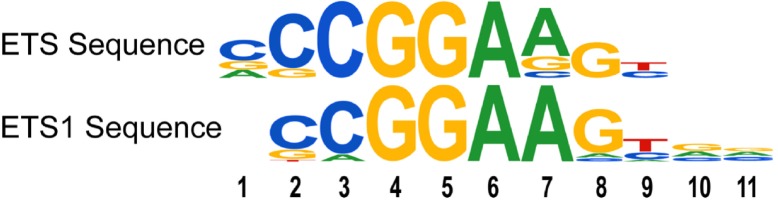
Mutant p53, p53 R273H, binds genomic sites enriched for ETS-related binding sites for ETS1 and GABPA The over-represented ETS-related sequences within genomic regions associated with p53 R273H binding peaks, are depicted as a sequence logo (ETS sequences). A sequence logo for the binding sites for ETS 1 is shown for comparison.

We examined the DNA sequences bound by p53 R273H by selecting a narrow DNA region for a single peak of binding for each promoter. Since many promoters had multiple peaks, the DNA sequence corresponding to the largest peak from each promoter was selected, with a peak width of 145 bases. We identified statistically significant over-represented 7mer sequences specific to p53 R273H peak regions. One set of over-represented sequences matched the transcription factor binding sites (TFBS) for ETS1 and GABPA (referred to as ETS Sequences). This is consistent with a similar analysis [12]. A sequence logo, shown in Figure 1, was used to summarize these conserved sequences and for comparison to the logo for the ETS1 binding site. The most prevalent and canonical sequence representing the ETS sequences, CCGGAAG, was highly associated with the p53 R273H binding peaks, found in 7% of p53 R273H promoter peaks, with a p-value of 10^−117^. This result is consistent with the known physical interaction between p53 R273H and ETS1 [12, 13] that in turn is presumed bound to ETS1 binding sites.

### p53 R273H preferred binding to bidirectional promoters

An interesting caveat of the motif analysis for sequences within p53 R273H binding peaks was the abundance of sequences matching ETS1 binding sites oriented in both directions, as if the sites were involved in regulating transcription in opposing directions. This type of configuration is consistent with bidirectional promoters; promoters that have overlap and mediate transcription bidirectionally. Bidirectional promoters are defined as promoter regions in which two genes transcribed in opposite directions have transcription start sites (TSSs) back-to-back within 1 Kb of each other [22]. They are over-represented in the human genome and comprise approximately 5% of promoter regions, and can direct transcription of coding and non-coding RNAs [23]. Genes controlled by bidirectional promoters are enriched for cancer-related genes and DNA repair genes [24]. Bidirectional promoters are enriched for certain TFBSs that include binding sites for GABPA and ETS1 [25]. GABPA has been shown to specifically bind bidirectional promoters and to play a significant role in regulating bidirectional gene expression [25]. Therefore, it was a very intriguing possibility that p53 R273H had a preference for binding bidirectional promoters.

We identified 1081 promoter regions within the human genome in which there were opposing gene TSSs oriented back-to-back with a distance between 0 and 1000 bases between them. The genes associated with these promoters were coding and non-coding, including miRNA genes. Of the 1081 bidirectional promoters, 177 were bound by p53 R273H (p-value = 10^−78^). This represented 22% of promoters that bound p53 R273H, and 16% of bidirectional promoter regions. p53 R273H was approximately 7-times more likely to bind a bidirectional promoter than a unidirectional promoter. Promoter regions that had two opposing TSSs oriented face-to-face with a distance of 0 to 1000 bases between TSSs were identified separately. These regions are not considered bidirectional promoters because the promoter regions while being somewhat close to each other, do not overlap. Remarkably, we found no statistically significant association between p53 R273H binding and these promoter regions (p-value=0.12). This suggested that the bidirectional configuration of TFBS, such as GABPA and ETS1 and the factors that bind those sites, might be directing the binding by p53 R273H.

### Preferred binding of p53 R273H independent of promoters

We wanted to know if the association between p53 R273H and putative ETS1/GABPA binding sites was dependent on the sites being within promoter regions where other factors bind and complexes form to promote transcription. We identified putative ETS1 and GABPA binding site locations, and because of their substantial overlap, combined the locations, and refer to them as putative ETS binding sites. We looked at regions with only a single ETS binding site compared to regions with only two sites within 1 Kb of each other; regions in which no promoter or other ETS binding site was within 2 Kb. The results in Table 1 show there to be a substantial and statistically significant association between p53 R273H binding sites and isolated single putative ETS binding sites. We gauged the propensity for p53 R273H to bind by assessing the fraction of sites to bind p53 R273H. The fraction of bound sites with single ETS sites was 0.065, with a p-value of <10^−300^. Remarkably, the addition of a single inverted ETS site increased the propensity for p53 R273H to bind by 3.7 fold (comparing 0.24 to 0.065). This substantial increase could result from coordinated or cooperative binding between multiple ETS 1 or GABP sites and p53 R273H. There was also a statistically significant association between p53 R273H binding and regions with direct repeats of two ETS sites.

**Table 1:** 

ETS sites	Number of site-containing regions	Regions with R273H binding	p-value	Fraction of sites bound by p53 R273H
ETS singles	11165	726	<10^−300^	0.065
ETS1 inverted dimers	324	78	10^−122^	0.241
ETS1 direct dimers	332	71	10^−107^	0.214

### p53 R273H binding correlates with ETS1 and GABPA binding

We used ChIP-Seq to determine locations of binding for ETS1 and GABPA within H1299 p53 R273H cells, and specific binding within promoter regions. Table 2 shows how the binding locations within promoters for ETS1 (571 promoters, Supplemental Table 2) and GABPA (1594 promoters, Supplemental Table 3) correlate with where p53 R273H binds, just as the putative ETS binding sites did. The binding sites for p53 R273H shows a statistically significant correlation with the sites bound by ETS 1 alone, GABPA alone, and both ETS1 and GABPA. However, we stress that the certainty that a promoter has no ETS 1 binding or no GABPA binding is low, thus it's possible that the association of p53 R273H binding with GABPA alone could be attributed to ETS 1 binding.

**Table 2:** 

Promoters	Number of promoters common with R273H binding	Number of promoters with either GABPA, ETS 1, or both bound	p-value	Fraction of GABPA-ETS1 bound promoters bound by p53 R273H
ETS1 binding only	83	284	10^−54^	0.29
GABPA binding only	146	1373	10^−36^	0.11
GABPA and ETS1 binding	103	287	10^−77^	0.36

### Propensity for p53 R273H to bind promoters correlates with the number of ETS motifs

**Figure 2:**
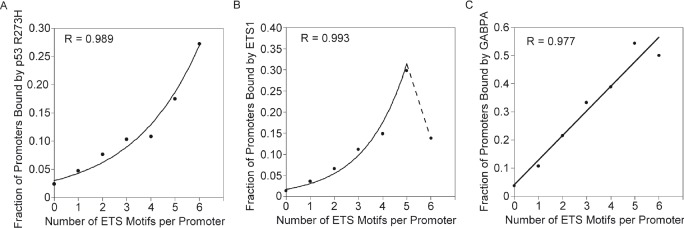
p53 R273H, ETS1 and GABPA, exhibit increased binding probability for promoters with higher number of ETS motifs A. Increase in probability of binding for p53 R273H versus an increase in the number of ETS motifs per promoter is shown in this plot. An exponential curve was the best fit for the data, with correlation coefficient, R, shown. B. Increase in probability of binding for ETS1 versus an increase in the number of ETS motifs per promoter is shown in this plot. An exponential curve was the best fit for the data, with correlation coefficient, R, shown for the first 6 points, with additional discontinuous point connected by a dotted line. C. Increase in probability of binding for GABPA versus an increase in the number of ETS motifs per promoter is shown in this plot. A linear curve was the best fit for the data, with correlation coefficient, R, shown. Standard error calculations for this data were not possible because the analysis is based on the intersection between three or two replicates and not on the average of signal.

We investigated the relationship between p53 R273H binding and multiple ETS binding sites further within promoter regions that included bidirectional promoters where multiple bidirectional ETS binding sites are prevalent. We identified all ETS motifs identified from the sequences with peaks of p53 R273H binding, as already illustrated in Figure 1. These sites represent a substantially broader and more inclusive set than the set of putative ETS binding sites analyzed in Table 1. The ETS motifs mapping offered a more precise and accurate position mapping than possible with binding data from ChlP-Seq. This is critical when motifs are closely spaced. The ETS motif sequences in both orientations were mapped and counted for each promoter region (1 kb) for all genes (24707 genes). The copy number for the ETS motifs, regardless of orientation, ranged from 0 to 6. There were a few promoters with more than 6 motifs, as high as 13, but were not included in the analysis because there were too few to analyze. The fraction of promoters with p53 R273H binding for each set of promoters with ETS motif copy number was determined and plotted. This fraction represents the probability for p53 R273H binding. The graph in Figure 2A shows an increase in probability of p53 R273H binding to promoters corresponding to an increase in the number of ETS motifs. The increase appeared to be exponential and an exponential function as shown had a better fit compared to a linear function. Thus, the data suggested a possibility of a coordinated or cooperative effect for binding relating to the ETS motif copy number. Analysis of ETS 1 binding as related to ETS motif copy number showed a similar relationship to the p53 R273H binding analysis. The graph in Figure 2B shows the probability of ETS 1 binding increasing with an increase in ETS motifs, but with a drop-off for promoters with 6 ETS motifs. We fitted an exponential function to the data for the first 6 points, omitting the point for 6 ETS motifs and showing a dotted line to connect that point. The deviation in binding propensity for p53 R273H and ETS1 for promoters with 6 ETS motifs does suggest a disconnection between p53 R273H and ETS1 binding, possibly because p53 R273H also associates with other factors that bind the ETS motifs. Analysis of GABPA binding (Figure 2C) showed a similar relationship with the number of ETS motifs as did p53 R273H and ETS1, though with no suggestion of an exponential relationship. For all three proteins, the increase in probability for binding generally increased in a proportional fashion with the number of ETS Motifs.

**Figure 3:**
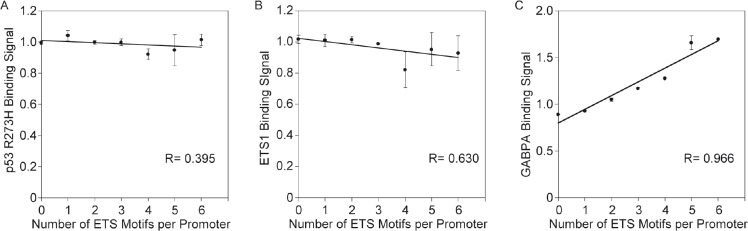
Binding signal for GABPA but not p53 R273H and ETS1 goes up with the number of ETS motifs per promoter A. The plot shows the average binding signal for p53 R273H does not increase with increase of number of ETS motifs per promoter. A linear curve fit and correlation coefficient, R, is shown. B. The plot shows the average binding signal for ETS1 does not increase with increase of number of ETS motifs per promoter. A linear curve fit and correlation coefficient, R, is shown. C. The plot shows the average binding signal for GABPA increases with increase of number of ETS motifs per promoter. A linear curve fit and correlation coefficient, R, is shown. For all plots, standard error bars are shown based on the relative average of replicate signals normalized to the mean of signal per promoter.

The results thus far indicate that the probability of binding for p53 R273H, ETS1 and GABPA increases with the number of ETS motifs, thus, it was reasonable to think that the amount of protein bound would also increase. We analyzed the relationship between average binding signal and number of ETS motifs. We would expect that if a greater number of protein molecules were bound to a single promoter at the same time that the binding signal based on the number of ChIP-Seq reads would be higher. Figure 3A shows a graph of relative average binding signal for p53 R273H plotted against the number of ETS motifs. A linear function was fitted to the data that shows a slightly downward trend in signal as the number of ETS motifs increases. We interpret this as evidence that there is no increase in the number of p53 R273H proteins bound associated with the additional ETS motifs.

Our analysis of ETS 1 binding signal showed similar results as for p53 R273H. Figure 3B shows a graph of relative average ETS1 binding signal versus the number of ETS motifs, revealing a slightly downward trend for ETS1 binding as the number of ETS motifs increases. Thus, p53 R273H and ETS1 follow a similar pattern and do not appear to increase in number of molecules bound per promoter despite the increase in binding sites. However, an increase was observed in our analysis of GABPA binding as shown in Figure 3C. The increase in relative average GABPA binding signal with the increase in number in ETS motifs supports the idea that multiple GABPA molecules are bound to the multiple ETS1 motifs within a single promoter. These studies therefore indicate that the promoters with higher number of ETS motifs have a better chance of being bound by ETS1, and p53 R273H (potentially through interaction with ETS1), and GABPA. Yet, for ETS1 and p53 R273H, it appears to be a zero-sum-gain in that the average signal does not go up.

### Coordinated binding of p53 R273H, ETS1 and GABPA to promoters with multiple ETS motifs

There are published studies that show ETS1 binding to inverted duplicate binding sites with cooperativity [26, 27]. ETS1 binding to single or duplicate binding sites appears to be a mechanism of regulation of ETS1's autoinhibition [27]. We hypothesized that if ETS1 is binding cooperatively to multiple binding sites and p53 R273H is doing the same through interaction with ETS1, then the spacing of these sites would be critical for binding. We asked if there was preferred binding of ETS1 and p53 R273H to promoters with a specific spacing of the ETS motifs. We used the promoters we already identified with multiple ETS motifs, but simplified the analysis by studying only promoters with 2 ETS motifs in an inverted configuration, either face-to-face, or back-to-back. The full spectrum for spacing between ETS motifs within promoters spanned distances from −1000 to +1000 bases. The negative distances represent distances between motifs that are face-to-face, while the positive distances are for motifs that are back-to-back. These distances are independent of where the ETS motifs are positioned within the promoters.

**Figure 4:**
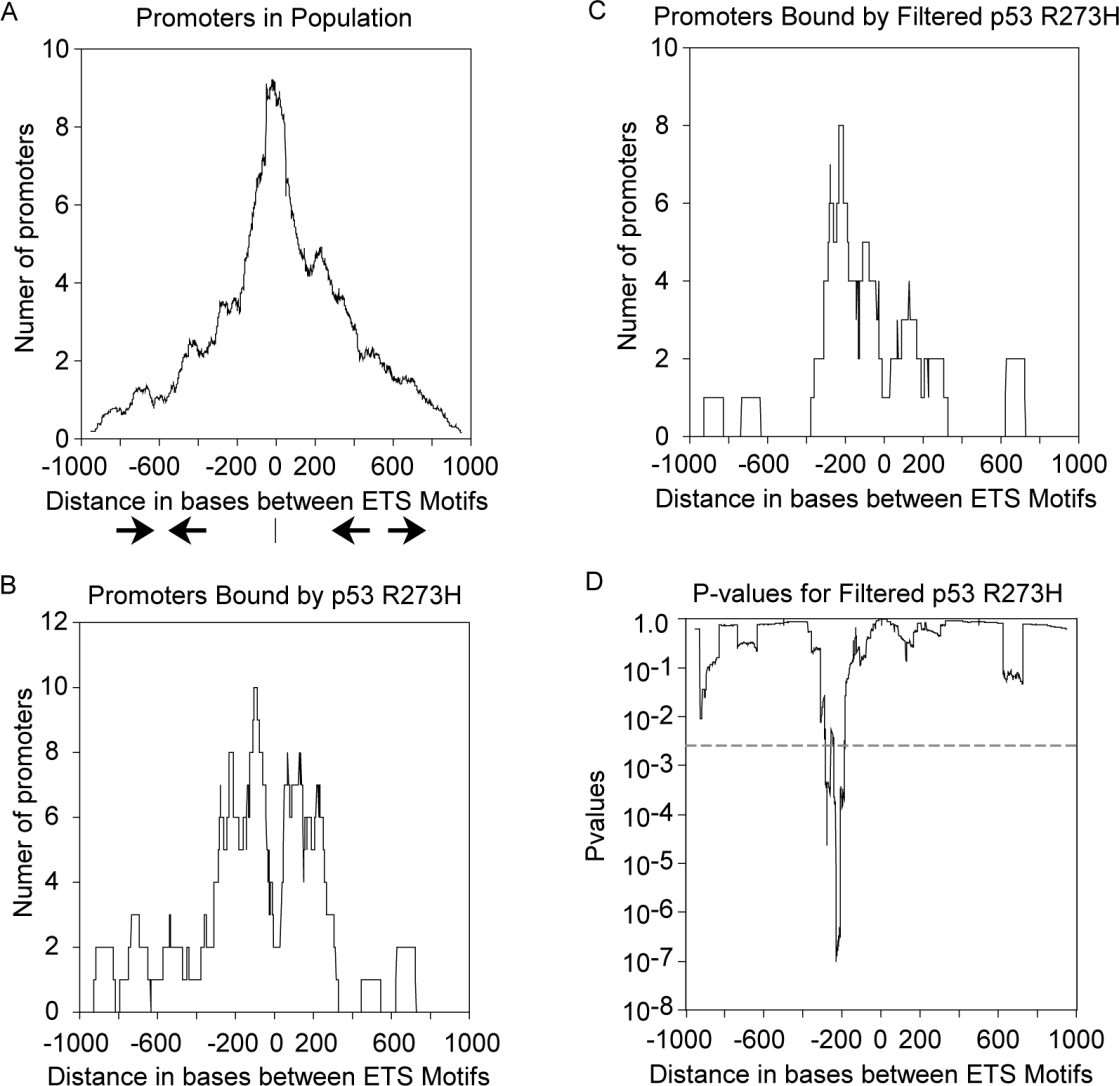
p53 R273H exhibits preferred binding to promoters with distantly spaced ETS motifs A. The distribution of distances between pairs of ETS motifs within all promoters is shown in a density plot. The distance range is from −1000 bps to + 1000bps based on the negative or positive distance between motifs oriented either face-to-face, or back-to-back, respectively. A diagram is shown with face-to-face arrows depicting the orientation of motifs on the negative side, while back-to-back arrows depict the orientation of motifs on the positive side. B. A density plot shows p53 R273H binding preference to promoters with distantly spaced ETS motifs in contrast to panel A. C. A density plot is shown for preferred binding by p53 R273H with high signal to distantly spaced ETS motifs. D. A plot is shown for p-values calculated for the corresponding p53 R273H binding in panel C in comparison to randomly sampled distributions. The dotted line corresponds to an alpha corrected for multiplicity of analysis.

The distribution of distances between motifs for all promoters with 2 inverted ETS motifs is shown in Figure 4A, scaled for direct comparison to p53 R273H binding data (the total sample size is ~24 times larger). Promoters with the ETS motifs closely spaced are predominant, shown by a sharp peak at zero distance and lesser shoulders diminishing toward −1000 and +1000. If p53 R273H has no preference for binding promoters with a particular distance between motifs, then the distribution of distances for promoters bound by p53 R273H should look like the distribution in Figure 4A. However, Figure 4B shows a conspicuous difference in that p53 R273H tends not to bind promoters with closely spaced ETS motifs as seen by the valley at the zero position. p53 R273H appears to have preference for binding promoters with ETS motifs spaced around 100 bases apart in both face-to-face and back-to-back configuration as shown by two peaks at around −100 and + 100. There's additional binding for promoters with ETS motifs ~200 bases apart at around −200 and +200 positions. This result could be because p53 R273H prefers to bind promoters with distantly spaced ETS motifs or because something prevents p53 R273H from binding to promoters with closely spaced ETS motifs. We also observed a distinct shift in the distance distribution for promoters bound by p53 R273H as we selected for higher binding signal. Figure 4C shows the distance distribution for promoters that bound p53 R273H in which we selected the top −50% of the original promoters for highest signal (425 promoters, see Supplemental Table 1). This stringent selection enriched for a subpopulation of promoters that bound p53 R273H with ETS motifs primarily oriented face-to-face with distances around 200 bases (a peak at the −200 position). We analyzed the data from Figure 4C statistically, comparing it to what would be expected by chance. A random sampling with replacement of the all promoters with inverted ETS motifs was performed 2500 times using a sample size equal to that in Figure 4C, generating data similar to the distribution in Figure 4A. We calculated p-values across the positions from −1000 to +1000 and plotted those in Figure 4D. The peak seen in Figure 4C can be seen as statistically significant with a p-value as low as 10^−7^, seen as a valley in Figure 4D. We interpret these results to indicate that p53 R273H shows preference for not binding promoters with closely spaced ETS motifs and a tendency to bind promoters with inverted ETS motifs spaced ~100 bps apart with a strong tendency toward sites spaced −200 bases apart face-to-face.

**Figure 5:**
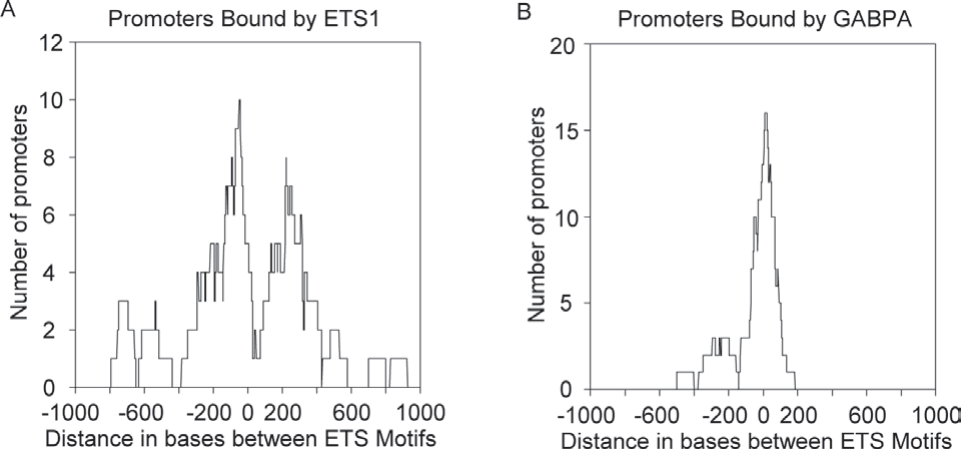
ETS1 exhibits preferred binding to promoters with distantly spaced ETS motifs while GABPA exhibits preference for promoters with closely spaced ETS motifs A. A density plot shows ETS1 binding preference to promoters with distantly spaced ETS motifs. B. A density plot shows GABPA binding preference for promoters with closely spaced ETS motifs.

We proposed that the most likely explanation for the distinct binding pattern for p53 R273H was because of its interaction with ETS1. Therefore, we analyzed ETS1 binding in the same manner to see if ETS1 also showed a similar distinctive binding pattern. Figure 5A shows the distribution for ETS motif spacing for promoters that bind ETS1 to be very similar to that for p53 R273H (seen in Figure 4B). The distribution pattern shows two peaks with a valley near zero, though it is slightly shifted to the right compared to that in Figure 4B. Two prominent peaks are around −50 and +200, with additional binding at positions around −200 and + 150. A selection for promoters with higher binding signal for ETS1 did not result in a shift in the distribution or change in pattern as found for p53 R273H (data not shown). We interpret these results to indicate that either ETS1 has a preference for binding ETS motifs distantly spaced or that something prevents its binding of promoters with closely spaced ETS motifs, or both.

We explored the possibility that GABPA also might show a preference for binding promoters with distantly spaced ETS motifs. Figure 5B shows the results for GABPA s binding preference and remarkably, it shows a binding preference that is the inverse to what ETS1 and p53 R273H showed. GABPA has a strong preference for binding promoters with closely spaced ETS motifs as seen by the sharp peak at the zero position, with a small peak around −200. It is striking that GABPA binds where ETS1 tends not to bind, and it's feasible that GABPA binding to closely spaced inverted ETS motifs either blocks or reduces binding to these sites by ETS1 and p53 R273H, explaining the valleys in binding for ETS1 and p53 R273H at the zero position.

**Figure 6:**
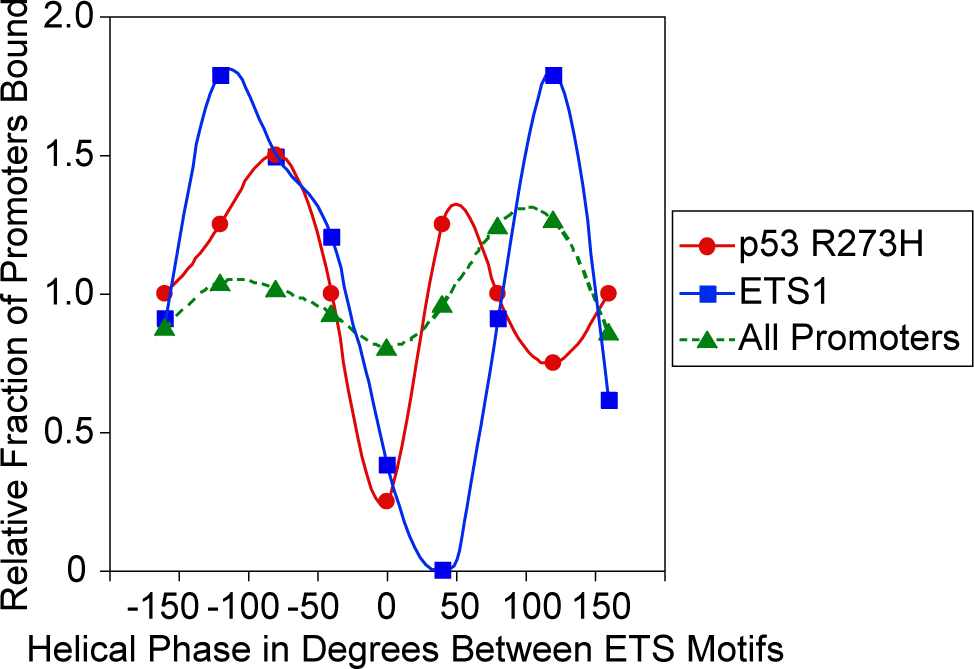
p53 R273H and ETS1 exhibit preferred binding corresponding to the helical phase between ETS motifs A plot is shown of the relative fraction of binding for p53 R273H (red) and ETS 1 (blue) as they relate to the helical phase between pairs of ETS motifs within promoters. The helical phase between pairs of ETS motifs for all promoters (green) is shown for comparison. Points are based the average relative binding for a window size of 40°. Smooth curves were fitted to the data.

Just as there can be a preference for binding of ETS1 and p53 R273H to promoters with a particular distance between ETS motifs, there may also be a preference for binding to promoters with the two ETS motifs positioned on the same face or different faces of the DNA helix. We determined the helical phase between ETS motifs from −180° to +180°, assuming a pitch of 10.4 bp/turn, however the actual pitch can vary as much as 1 bp per turn with supercoiling and nucleosome winding [28]. This variation would equal more than one entire turn over 100 bps, therefore, this analysis cannot provide an accurate determination of preferred phase for binding and is only intended to identify any potential pattern. The average number of promoters that bind ETS1 and p53 R273H normalized to the mean of total binding was plotted against the phase between the motifs, shown in Figure 6. Both ETS1 and p53 R273H appear to bind less to promoters that have motifs on the same face of the helix, indicated by the valleys around 0° in comparison to all promoters. There are peaks for p53 R273H and ETS1 binding for promoter with a phase between motifs from 50° to 120°.

## DISCUSSION

Analysis of binding by the GOF mutant p53 R273H, ETS1 and GABPA throughout the human genome and specifically in promoters regions has revealed a very intricate and novel relationship. The study of p53 R273H binding in regions with putative ETS1/GABPA binding sites and ETS motifs suggested that p53 R273H prefers to bind regions with multiple binding sites for ETS1/GABPA and possibly other factors in the ETS family. Evidence suggested the possibility of a cooperative effect in p53 R273H binding for regions with increasing number of ETS-related binding sites (Figure 2A). The known physical interaction between mutant p53 and ETS1 is the hypothesized explanation for this association with ETS-related binding sites.

A significant discovery stemming from the study of p53 R273H binding and associated DNA motifs was that bidirectional promoters were predominant among promoters that bind p53 R273H. Almost one fourth of the promoter regions bound by p53 R273H were bidirectional. This demonstrates the potential for p53 R273H to mediate novel bidirectional gene regulation and impact cancer genes, DNA repair genes, and miRNAs controlled by bidirectional promoters. The predominance of multiple ETS1 binding sites within bidirectional promoters is one possible explanation for the observed p53 R273H preferred binding.

ETS1 and p53 R273H showed a preferred binding to promoters with distantly spaced ETS motifs both face-to-face and back-to-back (~100–200 bp apart), and a distinct absence of binding to promoters with closely spaced ETS motifs (near zero bp) despite the fact that these promoters are by far the most abundant (Figure 3B, 4A). We conclude that p53 R273H exhibited the same binding preference as ETS1 because of its association with ETS1, at least in part. Analysis of promoters selected for having the most p53 R273H signal revealed that p53 R273H prefers to bind distantly spaced ETS1 motifs that are face-to-face as opposed to back-to-back, ~200 bp apart (Figure 4C). This posed the possibility that p53 R273H could exhibit preferred binding to a specific configuration of ETS1 bound to two distantly spaced ETS motifs. GABPA showed a strong preference for binding closely spaced inverted ETS motifs and not distantly spaced ETS motifs (Figure 5B). The binding preference of GABPA to closely spaced ETS motifs could possibly affect the binding of ETS1 to these sites.

**Figure 7:**
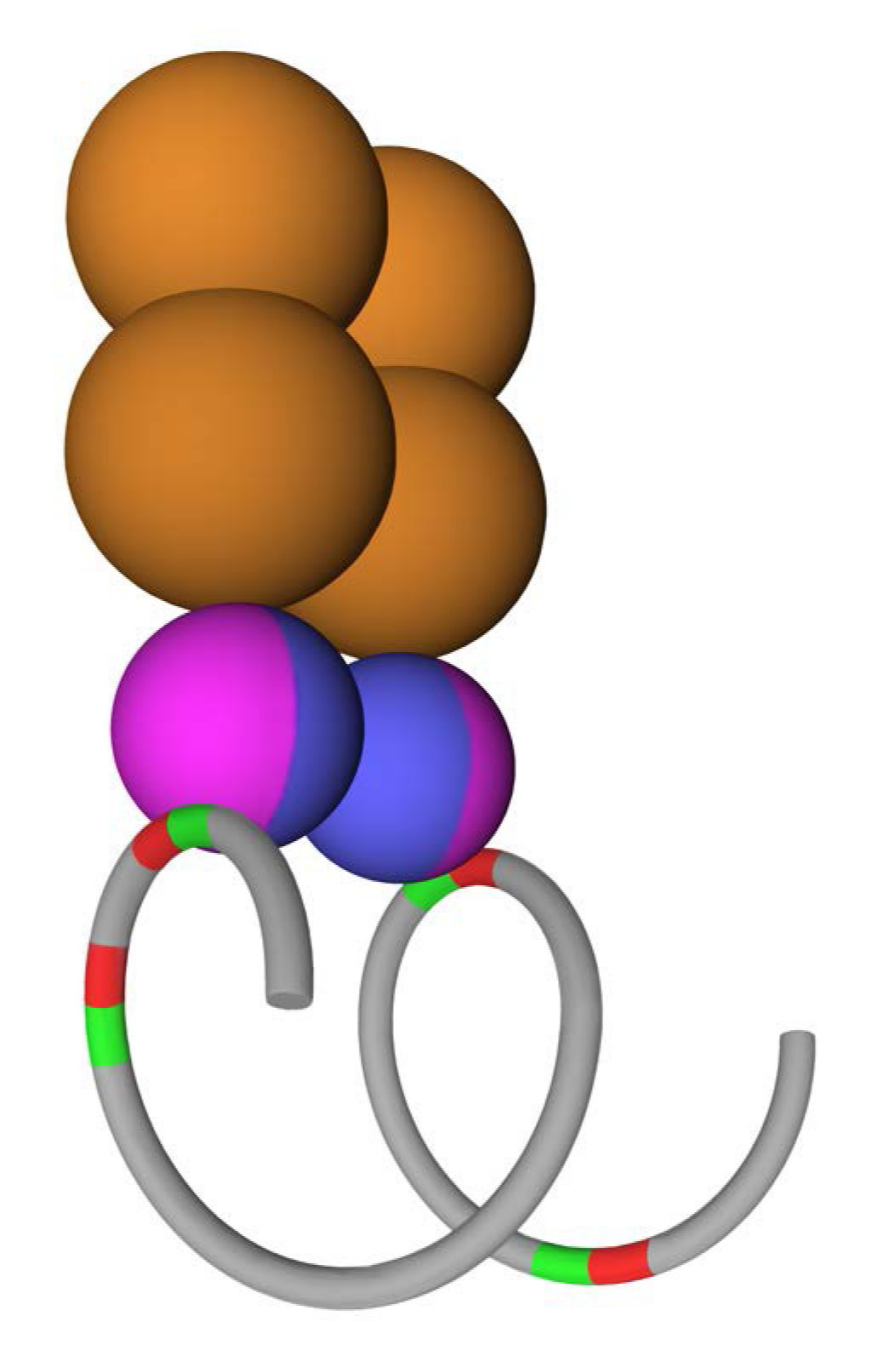
The DNA Loop Preclusion Model A proposed model is shown for ETS1 and p53 R273H binding to two inverted ETS1 binding sites. A DNA helix is shown in grey formed in a loop with four ETS1 binding sites shown in red and green (for orientation). Two ETS 1 monomers shown in lavender and blue are bound as a homodimer to two ETS1 binding sites. A p53 R273H tetramer is shown in orange bound to the ETS1 homodimer. Two ETS 1 binding sites are left unbound.

One of the most unexpected results from our studies was that there was no increase in signal for either ETS1 or p53 R273H binding for promoters with more ETS motifs, yet the probability of binding went up substantially. Thus, we suggest that regardless of the number of proteins for ETS1 and p53 R273H bound per promoter, which could be as few as one, that this binding precludes more proteins from binding despite the presence of additional binding sites. We propose that DNA forms a loop to bring two ETS sites together for binding. Our proposed model, the DNA Loop Preclusion Model, illustrated in Figure 7, accounts for our observations and conclusions. DNA containing 4 ETS1 binding sites in bidirectional orientations (as an example) forms a 360° loop so two sites are juxtaposed and anti-parallel. These two sites are each cooperatively bound by an ETS1 protein as part of a homodimer. The phase between these two ETS1 sites is 90°, though an actual preferred phase could not be accurately determined from our study. As a simplification, a single mutant p53 tetramer is shown bound to the ETS 1 homodimer, though we suggest that either one or two tetramers could be bound. The higher number of ETS 1 binding sites provides greater opportunity for ETS1 to bind, but once this loop structure forms, we propose that it precludes binding in this region of additional ETS1 and p53 R273H proteins. Our observations for the binding of GABPA to closely spaced ETS sites and the increasing signal with more ETS sites do not fit the DNA Loop Preclusion Model. We note that this model is quite similar to that already proposed from structural studies for ETS1 binding to widely spaced DNA binding sites [29]. In that structural model, two ETS1 proteins are cooperatively bound as a homodimer to two unconstrained anti-parallel DNA binding sites. The two sites are close to being in-phase, which would be equivalent to being on the same face of a DNA helix. Another structural study for two ETS 1 proteins bound to closely spaced binding sites has the sites 83° out of phase [26].

The proposed DNA loop in Figure 7 could easily be the result of DNA wrapped around a nucleosome core. One loop around a nucleosome core encompasses ~87 bps, which is close to some of the distances between ETS sites bound by ETS1. However, other distances between ETS sites bound by ETS1 are not consistent with a single loop around a nucleosome core. The preferred binding of p53 R273H to ETS sites ç200 bp apart would require an internucleosomal interaction.

While the association of GOF mutant p53 to ETS1 provides a substantial accounting for where GOF mutant p53 binds and acts on the genome, our results suggest that GOF mutant p53 also associates with other factors that bind DNA. A factor of the ETS family known to bind GOF mutant p53 that we did not account for is ETS2 [12], and so further study of the ETS family of proteins is warranted.

We have proposed what p53 R273H is doing in the cell in part through its binding to promoters, but it remains to be determined how this would alter gene expression. We speculate that p53 R273H may promote formation of the proposed loop structure in Figure 7, or stabilize it, thus increasing transcription from these bound promoters, possibly bidirectionally. The binding of p53 R273H to promoters might also result in chromatin alteration leading to gene activation. These possibilities represent intriguing directions for future studies.

## MATERIALS AND METHODS

### Cell lines, genes, promoters, transcription factor binding sites (TFBS), and DNA sequence data

Cell lines used were the human lung cancer cell line, H1299 (p53 null), transfected with vector alone (H1299 Vector), and transfected with vector expressing p53 R273H (H1299 p53 R273H), as previously described [30].

Data for gene mapping, promoter mapping and promoter sequences, and conserved TFBS (Transfac data) mapping, were provided by the UCSC Genome Brower, assembly GRCh37/hg19 [31]; http://genome.ucsc.edu]. All TFBSs were used in analysis without score selection. Promoter regions were defined as the 1 kb of DNA sequence immediately upstream of each gene's transcription start site (TSS). For genes with multiple start sites, only the most 5′ TSS was used.

### ChIP-Seq and ChIP-QPCR analysis

Chromatin immunoprecipitations (ChIP) were performed as previously described [32]. Briefly, 4.5×l0^6^ cells were plated per ChIP. Cells were cross-linked with 2% formaldehyde, and cross-linking was stopped using 0.2M glycine. Extracts were sonicated and immunoprecipitated using antibody to the protein target and Protein A agarose. Immune complexes were washed and cross-linking was reversed at 65°C overnight. DNA was digested with RNase A and proteinase K, phenol/chloroform extracted, and ethanol precipitated. All Abs were from Santa Cruz Biotechnology. ChIP for p53 R273H used Abs DO-1 (sc-126) and FL-393 (sc-6243); ChIP for GABPA used Ab GABPalpha (sc-22810); ChIP for ETS1 used Ab Ets-1 (sc-350); negative controls used normal mouse IgG (sc-2025) or normal rabbit IgG (sc-2027).

High-throughput DNA sequencing was performed at the Donnelly Sequencing Centre, University of Toronto, using the Illumina HiSeq system, single-end reads, and ELAND2 alignment. Alignment and genomic positioning for forward and reverse strand peak data based on their offset was performed by local optimization of the correlation coefficient between forward and reverse strand data, particularly needed for peak clusters. The ArrayStar QSeq Peak Finder was used for general peak finding.

There were three replicate ChIPs each for the H1299 p53 R273H with p53 Ab and negative control, H1299 Vector with p53 Ab. An additional negative control used H1299 p53 R273H and IgG. There were two replicates each for the ETS1, GABPA, and negative control IgG ChIPs. ChIP-Seq data were normalized to the mean total aligned reads per kb. The negative controls were used to assess a false discovery rate for binding data, which was set at ≤0.005. Final peak and promoter determination for binding was accomplished by determination of the intersection between replicates. This means that the genomic regions positive for binding are determined non-parametrically, and not based on the average of replicate signal. A small number of peaks and associated promoters showed to be positive for binding in all ChIP-Seq data including negative controls, and thus, attributed to bias, which were removed from further data analysis.

### Motif analysis

Over-representation of 7mer sequences was determined based on representation of each possible sequence within p53 R273H-bound 1 kb promoters and within all 1 kb promoter regions. P-values were calculated based on the hypergeometric distribution. Sequences were matched to conserved TFBSs using TomTom software [33, 34] with a maximum false discovery rate (FDR) of 0.01. Thirteen-base sequences with 2–4 additional bases flanking each side of the identified 7mers were used for the generated sequence logos. Sequence logos were generated with the assistance of WebLogo [35].

### Data analysis

The hypergeometric distribution was used for determining p-values for association between putative ETS sites and peaks of p53 R273H binding, ETS1 or GABPA binding sites and p53 R273H binding sites, and p53 R273H bound promoters and bidirectional promoters. In the case of bidirectional promoters, a promoter region was counted only once despite it encompassing two promoters.

The number of promoters bound by p53 R273H, ETS1 and GABPA for promoters with varying distances between ETS motifs (Figure 5 and 6) was determined for 100 bp windows with a 1 bp sliding window from −1000 to +1000 to generate density plots for the precise and accurate locations of peaks.

Analysis for Figure 4 was based on the average of signal for each category of promoters with ETS motif copy number, normalized to the mean signal per promoter within each replicate.

The statistical analysis for Figure 5D was based on the generation of 2500 runs of random sampling with replacement based on the sample size equal to that for the p53 R273H bound promoters in Figure 5C. The sum of promoters for a 100 bp window as already described at each bp position generated a series of 2500-point normal distributions. A normal distribution function was fitted to the data for each position and a p-value determined for each position from −1000 to +1000. Zero-inflated data that would not be normally distributed was only present at the very ends of the distribution, near -1000 and +1000, and did not affect interpretation.

## Supplementary Tables


